# Process intensification of continuous-flow seATRP by a sonicated multi-reactor setup

**DOI:** 10.1039/d3re00235g

**Published:** 2023-06-01

**Authors:** Suqi Zhang, Tanja Junkers, Simon Kuhn

**Affiliations:** a KU Leuven, Department of Chemical Engineering Celestijnenlaan 200F 3001 Leuven Belgium simon.kuhn@kuleuven.be; b Polymer Reaction Design Group, School of Chemistry, Monash University 19 Rainforest Walk, Building 23 Clayton VIC 3800 Australia

## Abstract

Simplified electrochemically mediated atom transfer radical polymerization (seATRP) is a versatile technique for synthesizing polymers with precise control and complex architecture. Continuous-flow seATRP has recently been realized by using a sonicated microreactor but still faces limitations such as relatively low conversion and difficulties in synthesizing polymers with high molecular weight. Herein, a novel multi-reactor setup is demonstrated. By tuning the currents applied to different reaction stages in the setup, 90% conversion can be achieved while maintaining relatively low dispersity (<1.35). Meanwhile, the unique design enables a wider processing window for sonication due to greater viscous attenuation in the second reactor, thus largely addressing the problem associated with high viscosity during the synthesis of high molecular weight polymers. The developed setup also offers an alternative strategy for future scale-up of continuous-flow seATRP.

## Abstract 2

A sonicated electrochemical multi-reactor setup is demonstrated for continuous-flow seATRP.

## Graphical abstract

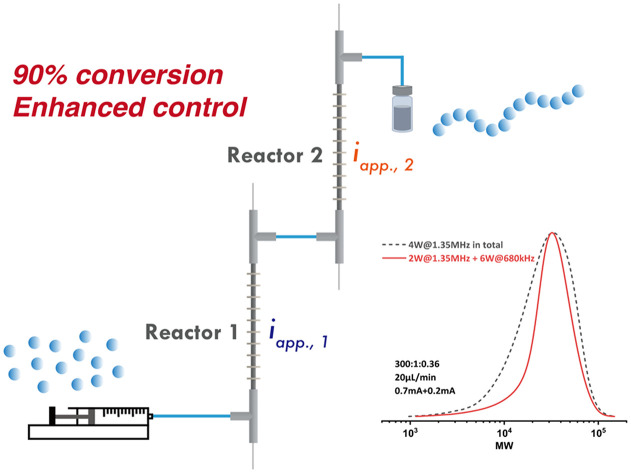

## 1 Introduction

Since its discovery in 1995, atom transfer radical polymerization (ATRP) has demonstrated its power through its application in many fields. Polymers with more complex compositions (*e.g.*, block,^1^ gradient copolymers^2^) or architectures (*e.g.*, brush,^3–5^ star^6,7^ and network^8^) have been successfully prepared by ATRP.

ATRP is a redox-active transition metal complex (commonly Cu/L, L: ligand) mediated catalytic process, which forms a dynamic equilibrium between dormant (alkyl halide initiator R–X and polymer P_*n*_–X) and active species (P_*n*_˙). A lower oxidation state catalyst (Cu^I^/L) reduces (activates) the dormant polymeric chain and itself is oxidized to its high oxidation state (Cu^II^/L). The activated polymeric chain can temporarily propagate, or get deactivated back to the dormant state by reacting with Cu^II^/L. ATRP has become more universal in its application thanks to some key advancements and the combination with externally applied stimuli. Many interesting processes have already been demonstrated and investigated, *e.g.*, photo-,^9–12^ sono-,^13^ mechano-^14,15^ and eATRP.^16–18^ Among these novel approaches, eATRP (electrochemically mediated ATRP) is particularly interesting for its various advantages of *in situ* generation of active catalyst, real-time control of reaction rate, enhanced tolerance to oxygen, *etc.*

eATRP is typically performed in a three-electrode system, consisting of a cathode, an anode, and a reference electrode. Cu^II^/L is reduced at the cathode and converted to Cu^I^/L, allowing the ratio between the two species to be controlled by the applied potential (*E*_app_). Separation of the anode from the reaction mixture is required to prevent undesirable anodic side reactions such as the oxidation of activators (Cu^I^/L),^16,17^ which significantly increases the complexity of the reactor setup and hindered its further commercialization.

Hence, a simplified eATRP process (seATRP) is developed: by the introduction of an aluminum sacrificial anode, the polymerization can be performed in an undivided cell.^16^ Although seATRP still has limitations due to its electrochemical nature such as: (1) a large amount of supporting electrolyte must be added to reduce the cell voltage and maintain a reasonable current. (2) Electrode surface-to-volume ratio is limited and (3) the necessity of small inter-electrode volume.

**Fig. 1:**
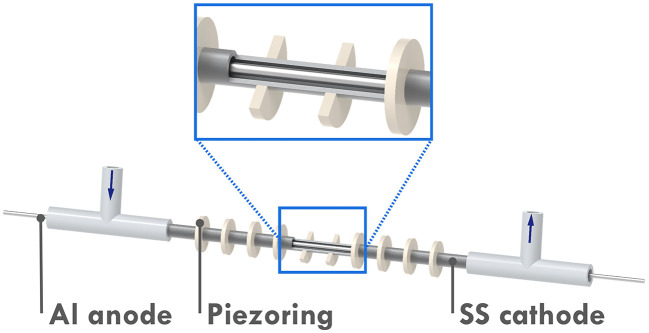
Schematic rendering of the seATRP microreactor setup.

Recently, a novel microreactor setup was reported by our group which enabled continuous-flow seATRP with the help of sonication.^19^ As shown in Fig. 1, the microreactor mainly consists of a 10 cm-long stainless-steel tube (od = 3 mm, id = 2.1 mm) and an aluminum wire, both act as electrodes in the electrolysis. The Al wire is positioned by T-junctions at both ends, providing a reactor volume of 268 μL. 10 piezoelectric rings (id = 3 mm, od = 8 mm, 1 mm thick) are fitted to the tube periodically with a space of 5.5 mm. UV-cured glue is applied to keep the rings in place and to seal the tiny gap between the rings and the tube. By taking advantage of the sonicated microreactor and continuous processing, the aforementioned drawbacks of traditional seATRP can be overcome largely. After adding sufficient excess ligand to suppress the adverse effect of released Al^3+^, a high monomer conversion of 75% was achieved in 27 min, compared to several hours needed in batch. In addition, no supporting electrolyte was required, which also significantly reduced the cost of downstream processing. As the reaction in a microreactor was conducted in a galvanostatic manner, the potential of the working electrode could not be precisely controlled. Thus there was inevitably a certain amount of catalyst loss during the reaction through direct electrochemical reduction of Cu^I^/L or Cu^II^/L to Cu^0^.^18^ This was even more obvious when operating at low flowrate (*i.e.*, long residence time). Therefore, the setup faces limitations when aiming for high conversions.

**Fig. 2:**
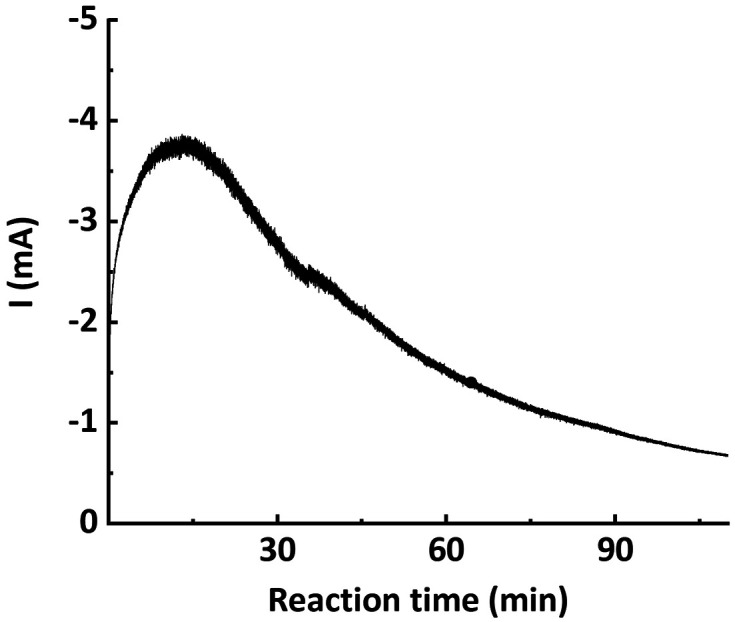
Chronoamperometry (*i*–*t* profile) of potentiostatic seATRP in batch.

From the chronoamperometry (*i*–*t* profile) of traditional potentiostatic seATRP conducted in batch reactor setups, it can be seen that the current drops exponentially over the course of the reaction, as shown in Fig. 2. This is presumably due to the fact that in the initial phase a relatively high current is needed to reduce the provided Cu^II^/L to Cu^I^/L to initiate the reaction, while in the later phase the current is only providing compensation for the formed Cu^II^/L due to termination. In previously reported galvanostatic seATRP successfully conducted in batch, several steps of different current values were applied to mimic the current profile recorded in a potentiostatic seATRP process.^16,18,20^ In a typical electrochemical microreactor setup, however, this operating pattern is difficult to realize as only one constant current can be applied. If the selected *i*_app._ is too low, not sufficient activator would be generated in the initial phase; while if the *i*_app._ is too high, then in the later phase the actual potential of the working electrode (cathode) would be so negative that it would favor the electroreduction forming Cu^0^, causing catalyst loss. These different optimum currents at different stages of the reaction make it difficult to identify a single “proper” current to apply.

**Fig. 3:**
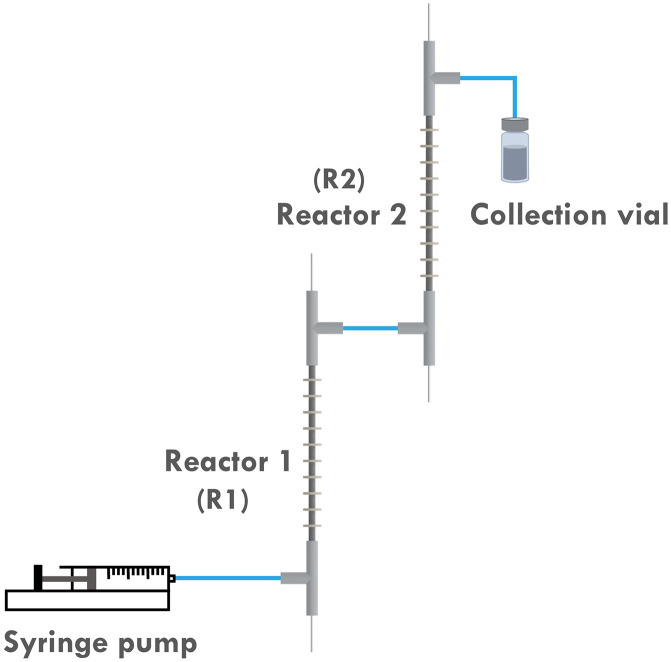
Scheme of the two-reactor setup.

To address this, the multi-reactor setup depicted in Fig. 3 is developed, which allows us to apply different currents at different stages of the reaction. This would in turn better approximates the original potentiostatic process. Furthermore, as the current applied to the second reactor (**R2**) is lower than that to the first reactor (**R1**), the entire setup is more energy-efficient. Previous research also indicated that it is difficult to scale up the developed reactor by simply elongating its length. This is attributed to the fact that even a slightly curved inner electrode can cause a short circuit when a single reactor is elongated too much. However, by employing a multi-reactor configuration, this issue can be circumvented as it allows for a facile scale-up strategy involving the integration of the required number of reactors.

## 2 Experimental

### 2.1 Materials

Methyl acrylate (MA, >99%), butyl acrylate (BA, >99%), acetonitrile (MeCN), copper(ii) bromide (CuBr_2_, 99%), tetrabutylammonium hexafluorophosphate (TBAPF_6_), and ethyl α-bromoisobutyrate (EBiB, 98%) were purchased from Merck. Tris[2-(dimethylamino)ethyl]amine (Me_6_TREN, 98%, TCI) was purchased from VWR.

Aluminum wire (1 mm) and stainless-steel tube (od = 3 mm, id = 2.1 mm) were purchased from Goodfellow. T-junctions (P633 Tee Assembly, ETFE) were purchased from IDEX Health and Science. Piezorings (SMR837T1411) were purchased from STEMINC. UV glue was purchased from Revell. The tube and the rod were both connected electrically to a potentiostat (Metrohm Autolab M101) *via* wires soldered on them. Coupled piezorings were connected to an E&I 2100L amplifier, which amplifies the sinusoidal signal generated by a Rigol DG1032 wave generator.

### 2.2 Continuous-flow seATRP procedure

Polymerization of MA was selected as the model reaction. 6 mL of MeCN, 6 mL of MA, 97.2 μL of EBiB, 0.795 mL of CuBr_2_/Me_6_TREN stock solution (0.1 M in MeCN), and 28.6 μL of excess Me_6_TREN to compensate dissolved Al^3+^ were added in a Schlenk flask, the dissolved oxygen was removed by 5 freeze-pump-thaw cycles. The degassed solution was then transferred to a syringe and pumped into the reactor setup using a syringe pump (Harvard Apparatus PHD Ultra). Above mixtures were adjusted for different [M] : [I] : [Cat.] ratios and different flow rates. Samples were collected after four residence times. The continuous-flow experiments were conducted in a ordinary laboratory fume hood.

### 2.3 Chronoamperometry of traditional batch potentiostatic seATRP

10 mL of MeCN, 10 mL of MA, 54.0 μL of EBiB, 774.89 mg of TBABF_6_, and 110.3 μL of CuBr_2_/Me_6_TREN stock solution (0.1 M in acetonitrile) were added in a Schlenk flask ([M] : [I] : [Cat.] = 300 : 1 : 0.03), then the dissolved oxygen was removed by 5 freeze-pump-thaw cycles. No excess ligand was added. The degassed solution was then transferred to a three-neck round-bottom flask and magnetically stirred in a nitrogen glovebox. A stainless steel tube (od = 3 mm, id = 2.1 mm, length exposed to the reaction mixture = 10 mm) was used as the working electrode and an aluminum wire coil (*d* = 1 mm) was used as the counter electrode. An Ag/Ag^+^ electrode was used as the reference electrode.

Cyclic voltammetry was first conducted to identify the potential to apply. This determined potential *E*_1/2_ was then applied and the reaction was performed at ambient temperature of around 23 °C and the change of current over reaction time was recorded.

### 2.4 Sonication conditions

Resonance frequencies of the solution filled reactor setup were first measured using an impedance analyzer (SinePhase 16777k). For each experiment, the signal frequency and amplitude were set on the signal generator and then amplified by an E&I 2100L amplifier. Two small ventilators (RS Components) were utilized to remove the heat generated by the piezorings.

For single-frequency sonicated experiments, two reactors were connected in parallel to one amplifier. While for two-frequency sonicated experiments, two sets of signal generator and amplifier were used separately to provide different sonication conditions to each of the two reactors.

### 2.5 Instrumentation

Galvanostatic/potentiostatic electrolysis was performed using a Metrohm Autolab M101 multichannel potentiostat.

Molecular weights (*M*_*n*_ and *M*_w_) and distributions (MWD) of the product poly(methyl acrylate) (PMA) were measured by gel permeation chromatography (GPC, SHIMADZU LC40) equipped with a differential refractive index detector (RID)and with THF eluent at 30 °C, 1.0 mL min^−1^ flowrate. A column set consisting of two PSS SDV linear analytical 5 μm 300 × 8 mm columns was used and calibrated using 10 polystyrene (PS) standards (*M*_*n*_ = 682–552 000 g mol^−1^). Mark–Houwink–Kuhn–Sakurada constants of relevant polymers were applied to correct measured molecular weights (PS: *K* = 0.0141, *α* = 0.7; PMA: *K* = 0.0102, *α* = 0.74).

The copper concentrations in the samples were measured using a PerkinElmer Optima 8300 Inductively Coupled Plasma Optical Emission Spectrometer (ICP-OES) equipped with an axial/radial dual plasma view, a GemTip Cross-Flow II nebulizer, a Scott double pass with inert Ryton spray chamber, a demountable one-piece Hybrid XLT ceramic torch, an echelle-based polychromator, and a two-dimensional, segmented CCD array solid state detector. All samples, calibration standards and quality control samples were diluted with 2 vol% HNO_3_. The system was calibrated with solutions containing 0.5 mg, 1 mg, 5 mg, 10 mg and 20 mg L^−1^ of Cu. A quality control sample was measured at the end of the analysis. Ga was added as internal standard to all samples but was applied only in case the quality control failed due to non-spectral matrix effects.

## 3 Results and discussions

### 3.1 Effect of applied current (*i*_app._) combinations

**Fig. 4:**
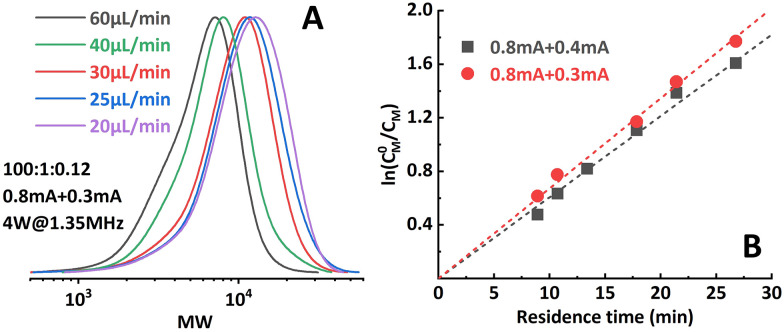
(A) Molecular weight evolution of poly(methyl acrylate) synthesized by the two-reactor setup. (B) Good linearity of the first-order kinetic plot when proper *i*_app._ combinations were selected.

**Fig. 5:**
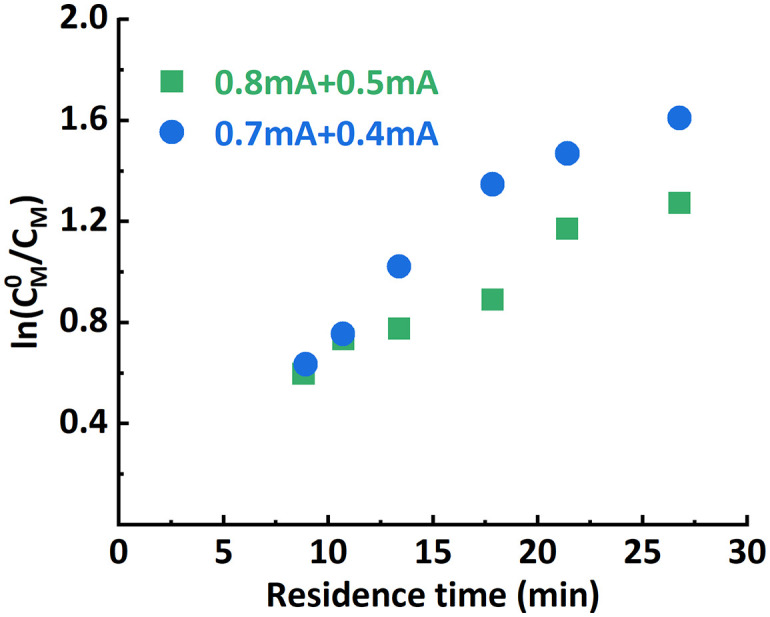
Non-linearity in the first-order kinetic plot when improper *i*_app._ combinations were selected.

The effect of *i*_app._ combinations applied to the individual reactor stages was investigated first. The polymerization results under different conditions are summarized in Table 1. Targeting a degree of polymerization (DP) of 100 ([M] : [I] = 100 : 1), it was found that when 0.8 mA and 0.3 mA are applied to **R1** and **R2**, respectively (“0.8 mA + 0.3 mA”), the molecular weight evolved well (Fig. 4A) and the dispersity index was low for all flow rates. A conversion of 90% could be achieved at a flow rate of 20 μL min^−1^ (corresponding to a residence time of 26.8 min), see entry 1 in Table 1. In comparison, the reaction stopped at around 75% conversion in the original single-reactor setup. Furthermore, as the *i*_app._ in **R2** was lower, less Al^3+^ was released from the sacrificial anode at the same residence time, which resulted in less excess ligand being required. Increasing the *i*_app._ on **R2** (entry 3, Table 1) resulted in lower conversion and a larger dispersity index. A similar effect was also observed when decreasing the *i*_app._ on **R1** (entry 4, Table 1). This can be explained by a poorer approximation of the potentiostatic experimental *i*–*t* pattern, which follows the results reported in galvanostatic seATRP in batch.^18,20^ The first-order kinetic plots showed good linearity when 0.8 mA + 0.4 mA or 0.8 mA + 0.3 mA was applied (Fig. 4B), while a non-linear behavior could be observed when 0.7 mA + 0.4 mA or 0.8 mA + 0.5 mA was applied (Fig. 5). We believe this is due to the fact that copper loss starts to have a substantial impact on the reaction mechanism.

**Table 1:** Results of the polymerization experiments in the developed two-reactor setup

Entry	[M] : [I] : [Cat.]	*i*_app._ (mA)	Flowrate (μL min^−1^)	Residence time (min)	Conversion (%)	*M*_*n*,GPC_	*M*_*n*,theo._	*Đ*
1	100 : 1: 0.12	0.8 + 0.3	20	26.8	90	9000	7900	1.32
2	100 : 1 : 0.12	0.8 + 0.3	30	17.8	74	7700	6600	1.35
3	100 : 1 : 0.12	0.8 + 0.4	20	26.8	86	8000	7600	1.35
4	100 : 1 : 0.12	0.7 + 0.4	20	26.8	86	8000	7600	1.37
5	300 : 1 : 0.36	0.7 + 0.3	20	26.8	56	16 000	14 700	1.58
6	300 : 1 : 0.36	0.7 + 0.2	20	26.8	66	18 900	17 200	1.60
7	300 : 1 : 0.36	0.5 + 0.2	15	35.7	61	17 700	16 100	1.61
8	300 : 1 : 0.36	0.6 + 0.2	15	35.7	66	16 300	17 200	1.60
9	300 : 1 : 0.36	0.8 + 0.3	25	21.4	49	11 800	12 700	1.92

**Fig. 6:**
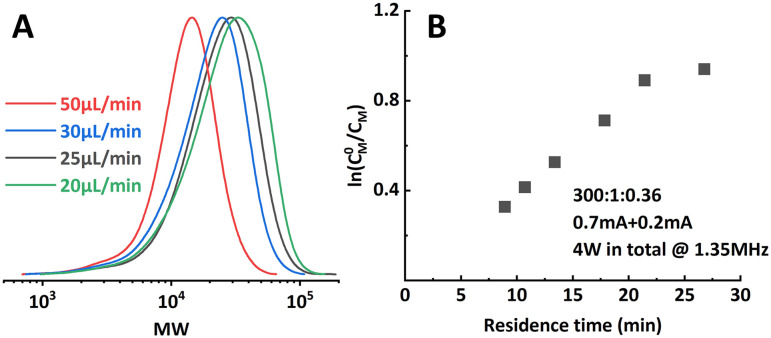
Limitations encountered when aiming at higher target DP. (A) Significantly wider MWD when operating at too low residence time. (B) Non-linearity in the first-order kinetic plot.

However, when targeting higher DP (300 : 1), the setup showed limitations in both conversion and dispersity control. Conversion stopped increasing at around 65% and a significant deviation from a linear first-order kinetic plot and wider MWD (Fig. 6; entry 5–6, Table 1) were also observed. Bimolecular terminated product formation was even observed in some extreme cases.

### 3.2 Effect of adding supplemental catalyst

**Fig. 7:**
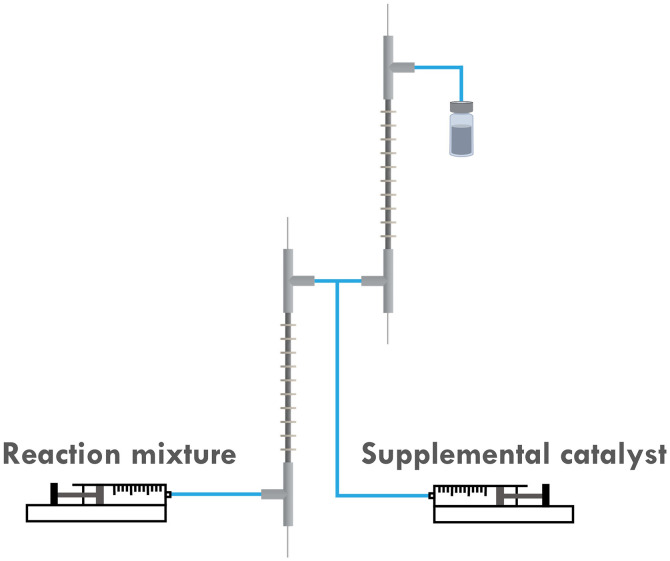
Scheme of the supplemental catalyst experiments.

**Fig. 8:**
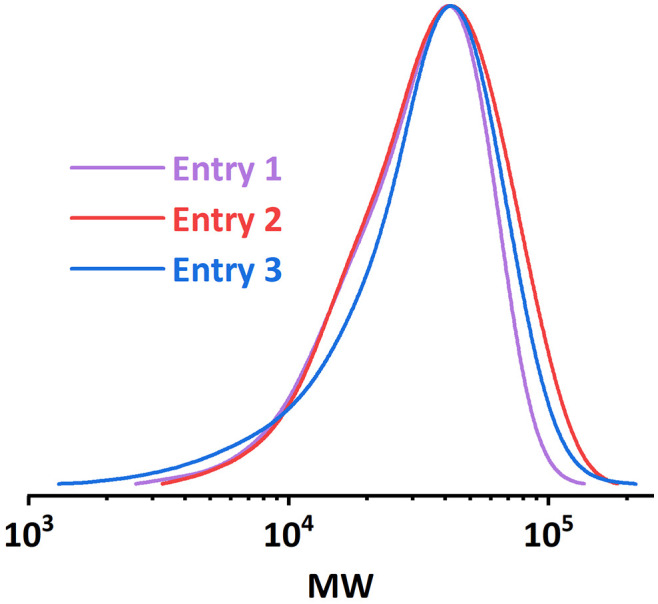
MWD comparison of entries in Table 2.

It was first presumed that the limitation encountered when targeting higher DP mentioned in the previous section was still caused by the loss of catalyst, while further tuning the *i*_app._ on both reactors did not show much positive effect on the results (entry 7–9, Table 1). Thus, the multi-reactor setup was modified by providing a new inlet for supplemental catalyst solution between the two reactor stages, see the scheme in Fig. 7. The feed rate of the catalyst solution was calculated to sufficiently compensate the catalyst loss in **R1**, which can be approximated by the data obtained from the single-reactor setup. The data is summarized in Table 2 and Fig. 8. It can be concluded that adding extra catalyst shows no obvious improvement in narrowing the MWD or increasing the conversion (entry 1, Table 2). Further increasing the feed rate of the supplemental catalyst solution also has no effect (entry 2–3, Table 2).

**Table 2:** Results of the supplemental catalyst experiments ([M] : [I] : [Cat.] = 300 : 1 : 0.36)

Entry	*i*_app._ (mA)	Flowrate (μL min^−1^)	Residence time (min)	Supplemental Cat. (%)	Conv. (%)	*M*_*n*,GPC_	*M*_*n*,theo._	*Đ*
1	0.7 + 0.2	20	26.8	30	62	17 900	16 200	1.54
2	0.7 + 0.2	20	26.8	40	66	20 600	17 200	1.62
3	0.8 + 0.4	15	35.7	40	62	19 400	16 200	1.71

### 3.3 Effect of sonication

During the polymerizations of higher target DP, a significant increase in pressure drop in the setup was noticed, which indicates a considerable higher viscosity of the reaction mixture. The associated larger attenuation of the acoustic streaming in the viscous solution could result in a lack of mixing,^21,22^ which would lead to a less controlled reaction. Thus we suspected that poor mixing was the reason for the observed lack of control.

**Fig. 9:**
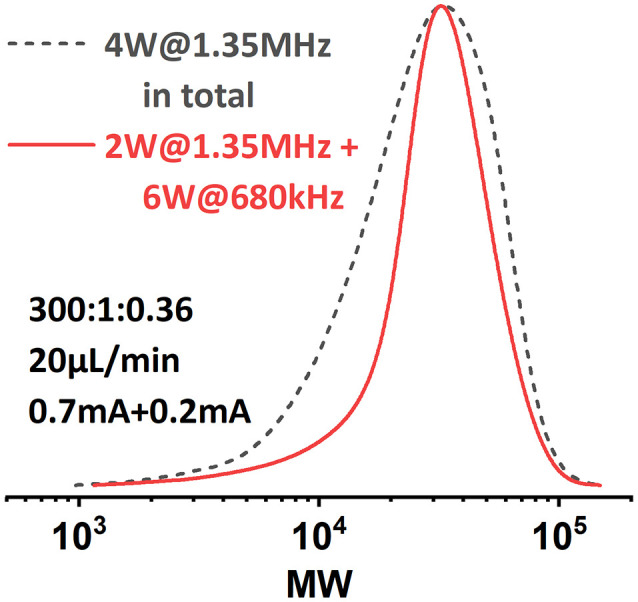
MWD comparison of PMA synthesized under different sonication conditions. [M] : [I] = 300 : 1.

By adjusting the sonication conditions in each reactor stage, it is possible to address this mixing problem in the reactors. We first tried to increase the sonication power applied on **R2** at the same frequency (entry 1, Table 3), but only little improvement in dispersity was achieved. When we shifted the operational frequency to a lower resonance frequency of the piezorings, a significant improvement in both conversion and dispersity was observed (entry 2, Table 3). Further increasing the sonication power also led to a slightly better result (entry 3, Table 3). The comparison of the GPC results is depicted in Fig. 9. Shifting to the lower actuation frequency in **R2** resulted in an improved penetration efficiency of the acoustic waves in the viscous solution, and thus an increase in the ultrasonic power applied to the reaction mixture. A higher power input will consequently result in a higher acoustic streaming velocity, and thus an enhanced mixing efficiency. Within the context of continuous-flow polymerization, this is typically translated to a narrower MWD.

**Table 3:** Results of the polymerization experiments targeting high DP with optimized sonication condition ([M] : [I] : [Cat.] = 300 : 1 : 0.36)

Entry	*i*_app._ (mA)	Sonication condition	Conversion (%)	*M*_*n*,GPC_	*M*_*n*,theo._	*Đ*
1	0.7 + 0.2	2 W@1.35 MHz + 6 W@1.35 MHz	65	19 900	17 000	1.55
2	0.8 + 0.3	2 W@1.35 MHz + 4 W@680 kHz	80	20 900	20 900	1.36
3	0.8 + 0.3	2 W@1.35 MHz + 6 W@680 kHz	79	23 900	20 600	1.33

This sonication condition was previously found to be detrimental to the seATRP process in the original single-reactor setup, presumably because of radicals generated by ultrasonic cavitation.^23^ Whereas, when target DP is higher, ultrasonic cavitation is largely suppressed by the increased viscosity of the reaction mixture in **R2** due to the limited cavitation bubble pulsation.^24^ Consequently, this offers us a wider processing window for sonication and greatly improves the system's capability of handling highly viscous polymeric liquids.

**Fig. 10:**
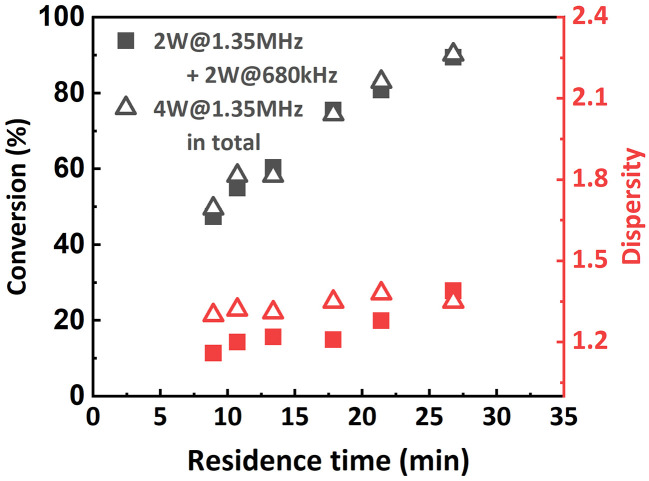
Molecular weight and MWD as a function of residence time. PMA was synthesized under different sonication conditions. [M] : [I] = 100 : 1.

Inspired by this, the effect of a reactor stage specific sonication condition was also investigated when targeting a lower DP of 100. **R1** was sonicated at 2 W with a frequency of 1.35 MHz, while **R2** was sonicated at 2 W with a frequency of 680 kHz. The results are summarized in Fig. 10. It was found that at higher flow rates (from 60 μL min^−1^ to 30 μL min^−1^), lower-frequency sonication in the later stage of polymerization led to narrower MWDs. However, when the flow rate was set to 20 μL min^−1^, the MWD was even wider than that from the original condition. We assume that in this case the viscosity of the reaction mixture in **R2** is not high enough to sufficiently suppress the ultrasonic cavitation, so when the residence time is too long (*i.e.*, longer sonication time), the ultrasonic cavitation induced radicals start to interfere with the ATRP reaction mechanism and eventually lead to a less controlled reaction. No significant effect on conversion was observed.

### 3.4 Catalyst loss

As mentioned above, one major disadvantage associated with galvanostatic electrolysis is the lack of accurate control over the working electrode potential. Specifically in the continuous-flow seATRP process, it directly causes copper catalyst loss due to over-reduction of Cu^I^ to Cu^0^ or direct electroreduction of Cu^II^/L to Cu^0^. To evaluate the copper catalyst loss in this multi-step galvanostatic electrolysis, ICP-AES was utilized to analyze the residual copper concentrations in the reactor effluent from certain different operational conditions.

The results are summarized in Table 4. When a lower current (0.4 mA) was applied on **R2**, at the same flow rate, the residual copper catalyst increased from 73% to 91% (entry 1 and 2, Table 4). Compared to the original single-reactor setup, the improvement was even more significant at the same residence time (entry 1, 4 or 3, 5, Table 4). This can be explained by a better approximation of a potentiostatic process, *i.e.*, in **R2** there is less electroreduction forming Cu^0^. It is likely that the process can be further intensified by more precise optimization of the applied current and residence time in different reactors.

**Table 4:** Catalyst loss measured by ICP-OES

Entry	*i*_app._ (mA)	Flowrate (μL min^−1^)	Residence time (min)	Residual Cu (%)
1	0.8 + 0.4	40	13.4	91
2	0.8 + 0.8	40	13.4	73
3	0.8 + 0.4	20	26.8	66
4	0.8	20	13.4	71
5	0.8	10	26.8	56

### 3.5 Durability and productivity

**Fig. 11:**
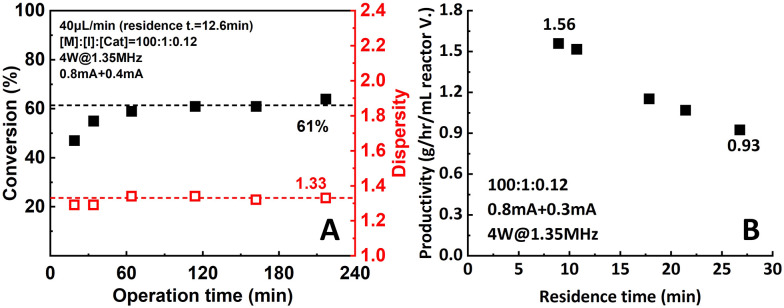
(A) Durability and (B) productivity of the two-reactor setup.

Durability and productivity are two very important parameters to assess microreactor setup performance. In this work, durability was tested by operating the reactor setup for a relatively long period of time (220 min, 16.5 residence times) under 0.8 mA + 0.4 mA of applied current. The reaction mixture ([M] : [I] : [Cat.] = 100 : 1 : 0.12) was pumped into the setup at a flow rate of 40 μL min^−1^. Samples were taken and measured to track the performance of the setup. As shown in Fig. 11A, after a stabilization period of around 4 residence times (60 min), conversion and dispersity reached a plateau of 61% and 1.33, respectively, and remain stable for the rest of the operational time.

Moreover, a significant increase in productivity was also observed (Fig. 11B). When the 0.8 mA + 0.3 mA current combination was applied, the highest observed productivity of 1.56 g h^−1^ mL^−1^ reactor volume was obtained at 60 μL min^−1^ flow rate. Compared to the data from the original single-reactor setup (1.08 g h^−1^ mL^−1^ reactor volume), a 47.2% increase was achieved. When aiming for higher conversion, the productivity still remained at 0.93 g h^−1^ mL^−1^ reactor volume at 90% conversion. This can be explained by the increase of apparent reaction rate due to the better control over the actual working electrode potential.

## 4 Conclusion

In this work, a continuous-flow seATRP process previously developed by our group was further intensified by expanding from a single-reactor setup to a multi-reactor setup. In the setup, different *i*_app._ can be applied in different stages of the reaction to better approximate the *i*–*t* profile of the batch seATRP process, consequently reducing the inevitable copper loss during the galvanostatic process.

The reaction rate in the novel multi-reactor setup is higher compared to the original. 90% conversion can be achieved at a 20 μL min^−1^ flow rate when proper current combinations are applied. Dispersity is maintained relatively low (<1.35) when proper reaction conditions are applied. The limitation associated with high viscosity commonly encountered when targeting higher DP was also addressed by tuning the sonication condition. As a lower *i*_app._ is needed in **R2**, this design brings additional merits of energy saving and less excess ligand consumption, making the process more energy-efficient and environmentally friendly. Excellent stability of the setup is demonstrated by running the setup for a relatively long operational time.

The design provides a facile strategy for scale-up by integrating more reactors. Moreover, by adding more reactors and tuning the *i*_app._ in each reactor, the batch *i*–*t* profile can be better approximated. Thus, it is possible that the continuous-flow seATRP process can be further developed. The near-quantitative conversion also paves the way for synthesizing block copolymers in a fully continuous operational manner in future endeavors.

## Author contributions

S. Z. contributed to the full extent of this work, ranging from the experimental setups, data acquisition, interpretation, analysis, to the writing of the manuscript. T. J. was involved in the discussions, some analyses as well as writing and reviewing the original draft. S. K. was involved in the discussions, writing, and reviewing of the original draft, and is responsible for funding acquisition and project administration.

## Conflicts of interest

There are no conflicts to declare.
